# Synovial Fluid Biomarker Profile After Intra-Articular Administration of Neosaxitoxin in Horses: A Feasibility Study

**DOI:** 10.3390/ani15162453

**Published:** 2025-08-21

**Authors:** Cristóbal Dörner, Néstor Lagos, Lissette Oyaneder, Bruno C. Menarim, Galia Ramírez-Toloza

**Affiliations:** 1 Escuela de Medicina Veterinaria, Sede Viña del Mar, Facultad de Ciencias de la Vida, Universidad Andrés Bello, Quillota 980, Viña del Mar 2520000, Chile; cristobal.dorner@unab.cl; 2 Programa de Doctorado en Ciencias Silvoagropecuarias y Veterinarias, Campus Sur Universidad de Chile, Santa Rosa 11315, Santiago 8820808, Chile; 3 Membrane Biochemistry Laboratory, Department of Physiology and Biophysics, Faculty of Medicine, University of Chile, Independencia 1027, Santiago 8380000, Chile; nlagosw@gmail.com; 4 Equestria Centro Médico Equino, Quillota 2260000, Chile; lissette.oyaneder@ug.uchile.cl; 5 Gluck Equine Research Center, Department of Veterinary Sciences, Martin-Gatton College of Agriculture, Food and Environment, University of Kentucky, Agricultural Science Center North, 1100 S Limestone S107, Lexington, KY 40508, USA; 6 Department of Animal Preventive Medicine, Faculty of Veterinary Medicine, University of Chile, Santa Rosa 11735, Santiago 6640022, Chile

**Keywords:** joint, neosaxitoxin, safety, cytokines, equine

## Abstract

Osteoarthritis (OA) is an inflammatory joint disease and the leading cause of musculoskeletal disability affecting human and veterinary patients. New therapeutics halting inflammation while preserving joint homeostasis remain a critical need. Voltage-gated sodium (NaV) channels regulate the pro-inflammatory response of macrophages in the synovium, the central driver of joint homeostasis. Neosaxitoxin (NeoSTX) is a phycotoxin that blocks NaV channels, conferring a unique potential to regulate joint inflammation. This study evaluated the safety of intra-articular administration of NeoSTX in horses. Sixteen horses were allocated into two groups (n = 8/each). One group received one intraarticular dose (20 µg/2 mL of saline) of NeoSTX into one tarsocrural joint, while the control group received 2 mL of saline (0.9% NaCl). No differences were observed between groups for systemic or local signs of inflammation, including objective measures of surface temperature and joint effusion. Concentrations of synovial fluid total nucleated and differential cell counts, total protein, glucose, calcium, and 23 cytokines/chemokines measured throughout this study did not differ between treatment groups. In this short-term study, intra-articular NeoSTX injection was shown to be well tolerated and likely safe. Ongoing studies should elucidate the role of NeoSTX in modulating synovial mechanisms of inflammation and its endogenous resolution.

## Simple Summary

Osteoarthritis is a common and painful joint disease in both humans and animals. In this study, we explored a new potential treatment using neosaxitoxin, a compound known to block sodium channels and potentially reduce joint pain and inflammation. We tested the safety of injecting the toxin directly into the joints of healthy horses. Sixteen horses were divided into two groups: one received a joint injection of neosaxitoxin and the other received a saline solution as a control. Over ten days, we monitored their health, behavior, joint inflammation, and changes in the synovial fluid. The results showed that neosaxitoxin did not cause any harmful effects, pain, or visible inflammation. Blood cells and proteins in the joint fluid remained normal and key inflammation biomarkers (like cytokines and chemokines) were unchanged. This indicates that injecting neosaxitoxin into horse joints is safe and well tolerated, making it a promising candidate for future treatments for joint inflammation and osteoarthritis.

## 1. Introduction

Osteoarthritis (OA) is the leading cause of musculoskeletal dysfunction in both veterinary and human medicine, translating into a significant health and financial burden [1,2]. Synovial inflammation is a central feature of OA development. Current OA therapies provide short-term improvement by inhibiting inflammatory mechanisms, which are, however, necessary for recovery of homeostasis [3,4]. Synovial macrophages play a crucial role in the homeostasis of joint tissues, including inflammation and tissue repair, and are essential for endogenous resolution of joint inflammation [5,6,7,8,9,10,11]. Successful treatment of OA is limited by a lack of therapeutics that can, instead of blocking inflammation, promote its endogenous resolution. Therefore, investigations of new and innovative therapies capable of the long-term regulation of joint inflammation while preserving mechanisms of joint homeostasis are a critical need.

In excitable cells of the nervous system and muscles, voltage-gated sodium (NaV) channels are essential to control membrane potential and, therefore, neuronal impulse propagation [12,13,14]. These ion channels have also been identified in non-excitable cells such as astrocytes, microglia, and macrophages. In addition to maintaining membrane potential, in immune cells, cations such as sodium and potassium are important in regulating calcium-mediated signaling pathways related to the immune response [15,16,17]. Recently, the presence of different NaV channels isotypes (NaV1.5 and NaV1.6) was demonstrated in equine macrophages, and the cellular localization of NaV1.6 was elucidated [18]. The role of NaV channels in these cells has been related to the regulation of cell migration, phagocytic clearance, endosomal acidification, and the promotion of pro-inflammatory responses [15,16,17,19]. From an inflammatory response point of view, NaV channels are associated with their ability to downregulate the canonical nuclear factor kappa B (NF-κB) pathway, known to significantly enhance the expression of inflammatory mediators in OA, such as interleukin 1 beta (IL-1β), tumor necrosis factor alpha (TNF-α), and inducible nitric oxide synthase (iNOS) [18,20,21,22].

Neosaxitoxin (NeoSTX) is a phycotoxin with a high capacity to block NaV channels. NeoSTX has been used as a local anesthetic and pain blocker agent in humans and veterinary patients when administered via epidural, periarticular, perineural, and subcutaneous injection [23,24,25,26]. In addition, its effect as an inflammation modulator has recently been studied [18], suggesting that blocking NaV channels with NeoSTX results in inflammation resolution and recovery of joint homeostasis. Therefore, defining the safety of intra-articular administration of NeoSTX, along with its roles in synovial cells and joint inflammation, is critical to harness its therapeutic potential. The objective of this study was to evaluate the feasibility of intra-articular administration of NeoSTX in horses. We hypothesized that NeoSTX would not elicit a detrimental response in a healthy joint. The findings of this study will support the development of proof-of-concept investigations to further elucidate the role of NeoSTX in modulating joint inflammation and promoting synovial tissue repair.

## 2. Materials and Methods

### 2.1. Experimental Design and Animal Selection

A prospective, randomized feasibility study was conducted to evaluate the safety of intra-articular administration of NeoSTX. Safety of the NeoSTX was established by performing serial behavioral assessments of the horses along with musculoskeletal evaluations. Clinical and molecular parameters that indicated the presence or absence of joint inflammation were evaluated. Accordingly, surface temperature was measured at the level of the tarsal–crural joint using thermography and synovial fluid samples were obtained for cytology and various biomolecule measurements (total proteins, glucose and calcium, cytokines, chemokines, and growth factors).

This study was conducted at the College of Veterinary and Animal Sciences of the University of Chile in the Departments of Animal Preventive Medicine and Equestria Equine Medical Center, Universidad Andrés Bello.

Sixteen tarso-crural joints were selected from 16 adult crossbred horses aged between 5 and 16 years old. Horses were stabled for a total of 40 days, including 10 days of adaptation, 10 days of clinical evaluation and SF sampling, and 20 days of physical evaluation. At the study endpoint, horses were euthanized as part of the research protocol (terminal animal study). The principles of the international care and use of animals in medical research, along with the Chilean national ethical guidelines for biomedical research involving animals, were strictly followed in the design of this study, and the protocol was approved by the “Institutional Committee of Animal Use and Care” (CICUA, certificate no. 23716–VET–UCH-1e). Informed consent was obtained from the horse owners enrolled in this study. Horses selected were clinically healthy and without any evidence of lameness associated with the tarso-crural joints, which was determined by a thorough musculoskeletal examination performed prior to the start of this study, including a radiographic exam. Horses presenting tarso-crural effusion or any clinical and/or radiographic abnormalities were excluded from this study.

**Figure 1:**
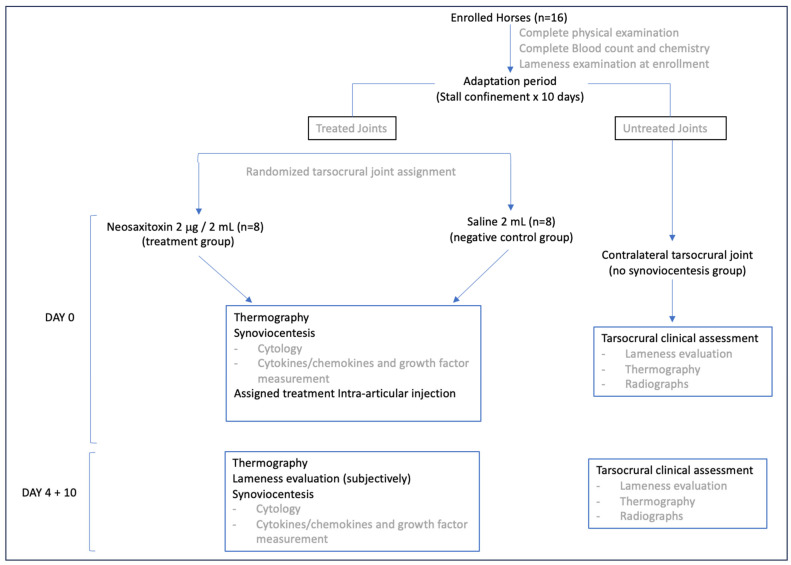
Schematic outlining study design.

Two groups of 8 horses each were established. Twenty micrograms of NeoSTX (diluted in 2 mL of 0.9% saline solution) was administered via intra-articular injection in the treatment group (N = 8). For the control group (N = 8), 2 mL of 0.9% NaCl was injected into the joint space. Both groups, treatments were administered at time 0 (initial). Local, systemic, and molecular effects were subsequently monitored through serial synovial fluid samples for a 10-day period (Figure 1).

### 2.2. Animal Maintenance

Horses were housed in 3 × 3 m stalls with shavings and fed free-choice alfalfa-based hay and fresh water provided ad libitum. Prior to the start of this study, the horses had a one-week adaptation period.

### 2.3. Neosaxitoxin Purification Procedure

Neosaxitoxin was purified by high-performance liquid chromatography (HPLC). The toxin was extracted from the cyanobacterial cultures (*Cyanobacteria aphanizomenon* sp.) as previously described by Lagos et al. (1999) [27]. The final toxin purity was confirmed by HPLC-MS and its concentration was standardized at doses of 10 µg/mL diluted in 0.9% saline solution.

The total 20 µg dose was established based on previous research that studied the effectiveness and safety of the doses, as well as the duration of the effect after NeoSTX administration in different species [23,24,25,26].

### 2.4. Synovial Fluid (SF) Sampling and Assessment

Synovial fluid samples were obtained aseptically with a 21G 1 1/2″ hypodermic needle, entering through the dorsomedial recess of the tarso-crural joint. A 2–4 mL synovial SF was obtained from each treated joint, divided into two tubes—one of them anticoagulant-free (Eppendorf^®^ Protein LoBind tubes) (Merck, Darmstadt, Germany) and another with EDTA (VacutainerTM) (Becton Dickinson, Franklin Lakes, NJ, USA)—at time 0 (baseline prior to treatment), 4 days post-treatment, and 10 days post-treatment. SF was analyzed according to its physical properties such as color, transparency, and viscosity. To eliminate cells from the sample (leukocytes, erythrocytes, and desquamative cells), samples without anticoagulant were centrifuged at 2000× *g* for 10 min. A small aliquot (200 μL) was immediately analyzed by a colorimetric method using a spectrophotometer (Chemo20^®^) (Shinova, Shanghai, China) to measure the total protein, calcium, and glucose. Additionally, total protein was also quantified by refractometry. Total protein was measured as an indicator of inflammation [28,29]. Regarding glucose and calcium, although not commonly quantified in SF evaluations, fluctuations can indicate inflammation/infection [30] and inflammation/cartilage degradation [15,31,32], respectively. The cell-free supernatants were stored at −20 °C for batch cytokine evaluation. SF samples were kept in EDTA tubes and analyzed within the first hour of collection using an automated hematology analyzer (Hemo plus 2900V^®^) (Shinova, Shanghai, China) to measure total nucleated cell count and the differential cell count of lymphocytes, monocytes, and neutrophils.

Horses were clinically assessed daily, recording heart rate (HR), respiratory rate (RR), temperature (T°), water and feed intake, and manure production, along with conducting a musculoskeletal examination, throughout this study. Clinical information gathered from treated joints was recorded for statistical analysis at days 0, 2, 4, 7, and 10 post-treatment. Joint inflammation was evaluated by the presence of synovial effusion and thermography, where changes in surface temperature were measured using a Flir One^®^ thermal camera at the level of the treated joints. Measurements were always taken in the same closed room, with a one-meter distance between the camera and the target area. Because horses had hair of different lengths in the area that could have directly affected the thermographic temperature measurements, hair over both tarso-crural joints was clipped at the beginning of the protocol. Additionally, the horses were subjected to daily lameness evaluations according to the lameness scale established by the American Association of Equine Practitioners (AAEP) [33,34]. Musculoskeletal examinations also included joint palpations, and passive flexion aimed at identifying synovial effusion or pain originating from the joint. Finally, after 30 days, and prior to euthanasia, new radiographic images were obtained to assess the presence of any bone changes that might indicate joint damage.

### 2.5. Cytokine Measurement

Synovial fluid concentrations of twenty-three analytes, including cytokines, chemokines, and growth factors, were assessed using Luminex^®^ xMAP^®^ technology (Thermo Fischer Scientific, Waltham, MA, USA). The multiplexing analysis was performed by Eve Technologies Corporation (Calgary, AL, Canada) using the Luminex^®^ 200™ system (Luminex Corporation/DiaSorin, Saluggia, Italy) with Bio-Plex Manager™ 6.2 software (Bio-Rad Laboratories Inc., Hercules, CA, USA). Twenty-three markers were measured in the samples using the Eve Technologies’ Equine Cytokine 23-Plex Discovery Assay^®^ Array (EQD23) as per the manufacturer’s instructions for use (MILLIPLEX^®^ Equine Cytokine/Chemokine Magnetic Bead Panel Cat. EQCYTMAG-93K, Millipore (Sigma, Burlington, MA, USA). The 23-Plex consisted of Eotaxin, FGF-2, Fractalkine, G-CSF, GM-CSF, GRO/KC, IFNγ, IL-1α, IL-1β, IL-2, IL-4, IL-5, IL-6, IL-8, IL-10, IL-12p70, IL-13, IL-17A, IL-18, IP-10, MCP-1, RANTES, and TNF-α. Assay sensitivities of these markers ranged from 0.70–255 pg/mL. Individual analyte sensitivity values were available in the MilliporeSigma MILLIPLEX^®^ protocol (Sigma, Burlington, MA, USA).

### 2.6. Statistical Analysis

The sample size for this study (n = 16; 8 per group) was determined using G*Power 3.1 software (Düsseldorf, Germany), employing an a priori power analysis to compute the sample size based on α, type II error, and effect size (f). A 95% confidence level, 0.05 significance level, and 80% power (1–β) with an effect size f of 0.3 were used.

For quantitative data, the Shapiro–Wilk test was used to assess data normality. Normally distributed data were first analyzed by a repeated measures ANOVA using Tukey’s post-hoc test. Quantitative data that were not normally distributed were log-transformed for the parametric analysis. Additionally, and given the nature of the data, a multivariate analysis was performed to detect potentially meaningful trends. A Generalized Linear Mixed Model (GLMM) and post-hoc pairwise comparison were applied, considering treatment, time, or their interaction as fixed effects and horses as a random effect. The 95% confidence intervals and size effects (squared Eta (η^2^)) were obtained from the linear model. Moreover, an exploratory principal component analysis (PCA) was performed to enrich the representation of the results obtained. All analyses were performed using R software version 4.0.2. Qualitative parameters were analyzed with the Pearson chi-square test. Significance was established at *p*-value < 0.05.

## 3. Results

### 3.1. Population Demographics

Sixteen crossbred adult horses (nine castrated males and seven females) with a mean age of 10.72 ± 3.93 years and an average weight of 389 ± 56.83 kg were included in this study. Synoviocentesis and intra-articular injections were successfully performed for all horses.

### 3.2. NeoSTX Did Not Induce Clinical Adverse Effects

**Figure 2:**
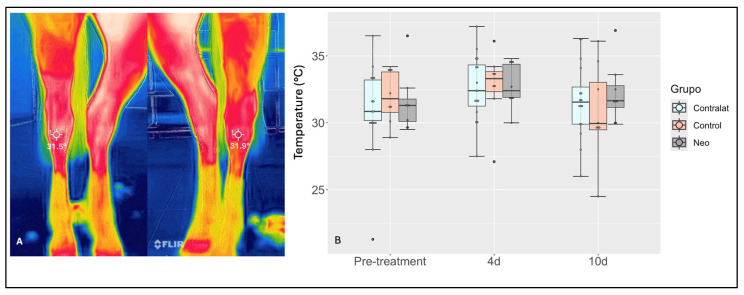
(**A**) Thermographic images of the tarso-crural joints of a horse at 4 days post-treatment depicting slight but non-significant differences in surface temperature (°C) as a result of either NeoSTX or saline injection. (**B**) Box plots of the results, with boxes representing interquartile ranges with a median and whiskers representing ranges. The contralateral tarso-crural joint was used as a control without treatment administration for clinical evaluation. A slight, insignificant increase in temperature was observed at 4 days after treatment administration, which returned to baseline by day 10. *p*-value = 0.05.

During the 30-day post-treatment evaluation, all horses presented normal behavior and no clinical or musculoskeletal abnormalities. Heart rate (*p* = 0.47), respiratory rate (*p* = 0.87), and body temperature (*p* = 0.17) were within physiological ranges and did not differ between groups throughout this study. Water and food intake were normal over the course of this study, and all horses maintained their body weight until the end of the protocol. No lameness was observed during the study period. As expected, imaging evaluation using pre- and post-study radiographs showed no changes associated with joint degeneration. Further, thermographic assessment of treated and contralateral joints showed a slight increase in surface temperature after the arthrocentesis procedure and treatment for both experimental groups (NeoSTX or saline). However, the increase was not significant (*p* = 0.11) and no differences were observed between NeoSTX- and saline-treated joints over time (*p* = 0.793) (Figure 2). Additionally, no effusion was observed in the tarsocrural joints of NeoSTX- or saline-treated horses, which was assessed visually and by digital palpation.

### 3.3. NeoSTX Did Not Alter the Cellular Profile of Synovial Fluid

**Figure 3:**
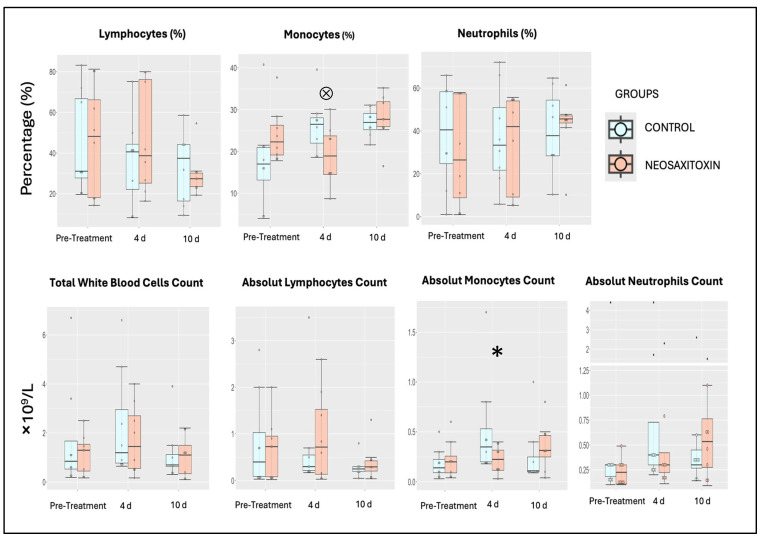
Values of the different cell populations present in equine synovial fluid. Box plots of the results over time for both groups, with boxes representing interquartile ranges with a median and whiskers represent ranges. Individual horses are shown as colored dots. **Top:** Relative cell counts of lymphocytes, monocytes, and neutrophils. **Bottom:** Absolute cell counts of total nucleated cells (leukocytes), lymphocytes, monocytes, and neutrophils over time in both study groups. * Statistically significant difference (*p* = 0.044). ^⊗^ This statistical trend was also observed in the relative monocyte population, though it was non-significant (*p* = 0.06). *p*-value = 0.05.

Assessments of total nucleated and differential cell counts in synovial fluid revealed similar patterns in NeoSTX- and saline-treated joints. In both groups, a transient increase in total leukocytes was observed at day 4 after treatment, which returned to baseline values at day 10 post-treatment (Figure 3). Differential cell count analysis revealed no differences in lymphocyte and neutrophil profiles between groups or within groups over the course of this study. However, in the NeoSTX group, the linear model revealed a statistically significant effect for the monocyte cell population at day 4 post-treatment administration (95% CI: 1.11 to 11.96), indicating downregulation compared to baseline values for the saline group. The model explained 12.4% of the total variance in the outcome (η^2^ = 0.124) (Table 1). The GLMM also showed this trend in the NeoSTX group at day 4 as being significant (*p*-value = 0.044) (Table S2, Supplementary Materials).

### 3.4. Intra-Articular Administration of NeoSTX Did Not Alter the Profile of Synovial Fluid Biomolecules

**Figure 4:**
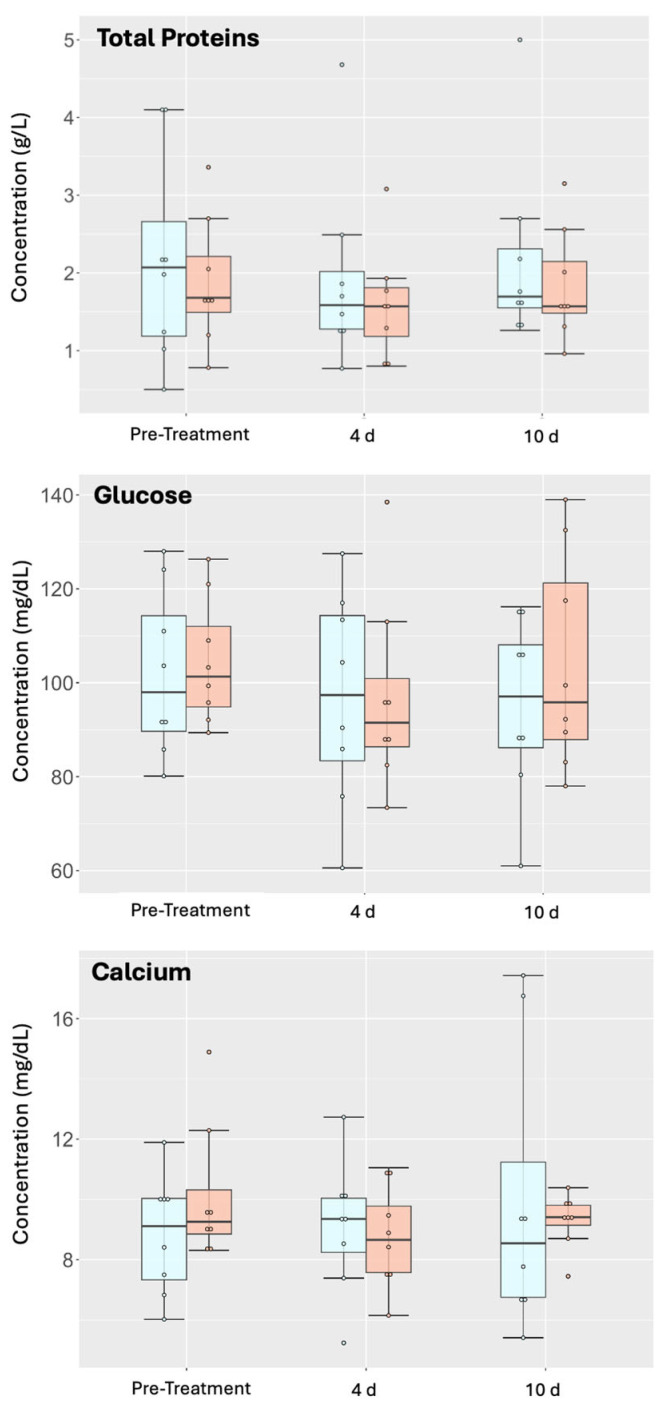
Evaluation of different biomolecules within synovial fluid over time. Measurement of total protein, glucose, and calcium at different time points, shown with the mean values, with whiskers representing standard deviations. No differences were observed between groups at different time points or within groups over time. *p*-value = 0.05.

Analyses of biomolecules present in the SF, such as total protein, calcium, and glucose, did not show significant differences between groups or within groups over time (Figure 4). There was a decrease in glucose concentration for NeoSTX-treated joints that failed to reach significance and returned to baseline by day 10. Glucose concentrations in saline-treated controls progressively decreased over time. There was a slight but insignificant decrease in total proteins for both groups at day 4 that returned to baseline by day 10; however, the absolute values remained within the reference ranges for the species. Finally, when evaluating the behavior of calcium over time, mean calcium concentrations decreased (*p* = 0.87) at 4 days after NeoSTX injection, slightly increasing by 10 days, while they only increased for the control group, yet these changes were not significant (*p* = 0.71).

**Figure 5:**
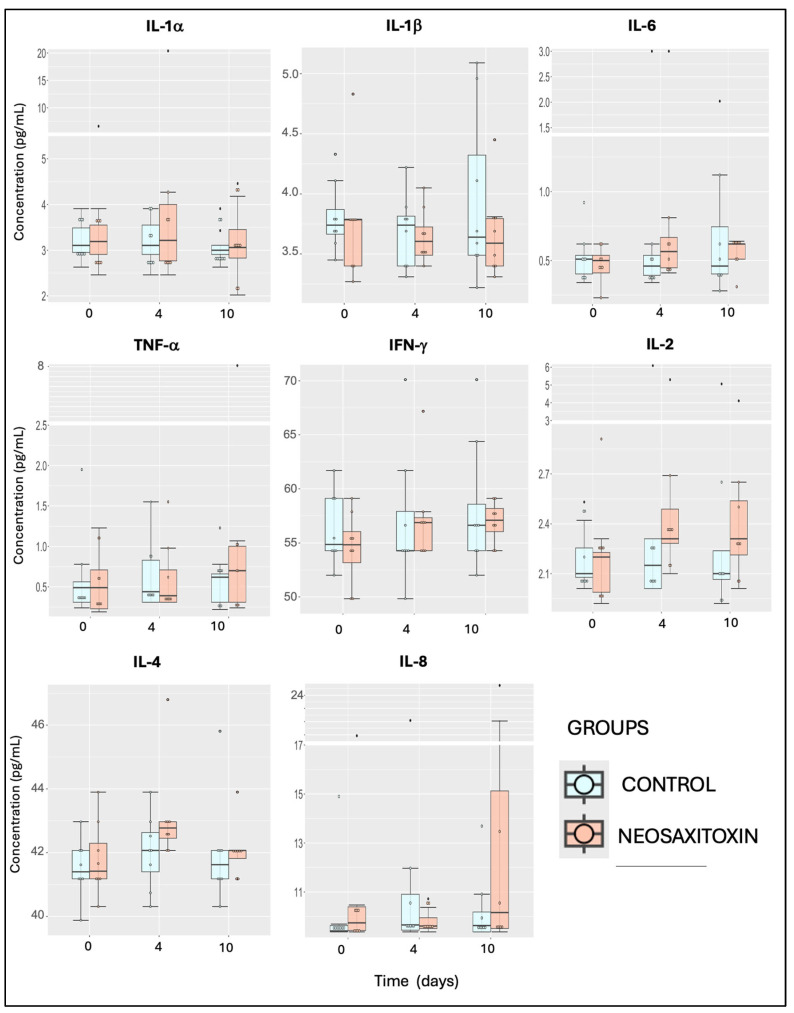
Synovial fluid cytokine concentrations from saline- and NeoSTX-treated joints over time. Boxes represent interquartile ranges with medians and whiskers represent ranges. Individual horses are shown as colored dots. No differences were observed between groups at different time points or within groups over time. *p*-value = 0.05.

**Figure 6:**
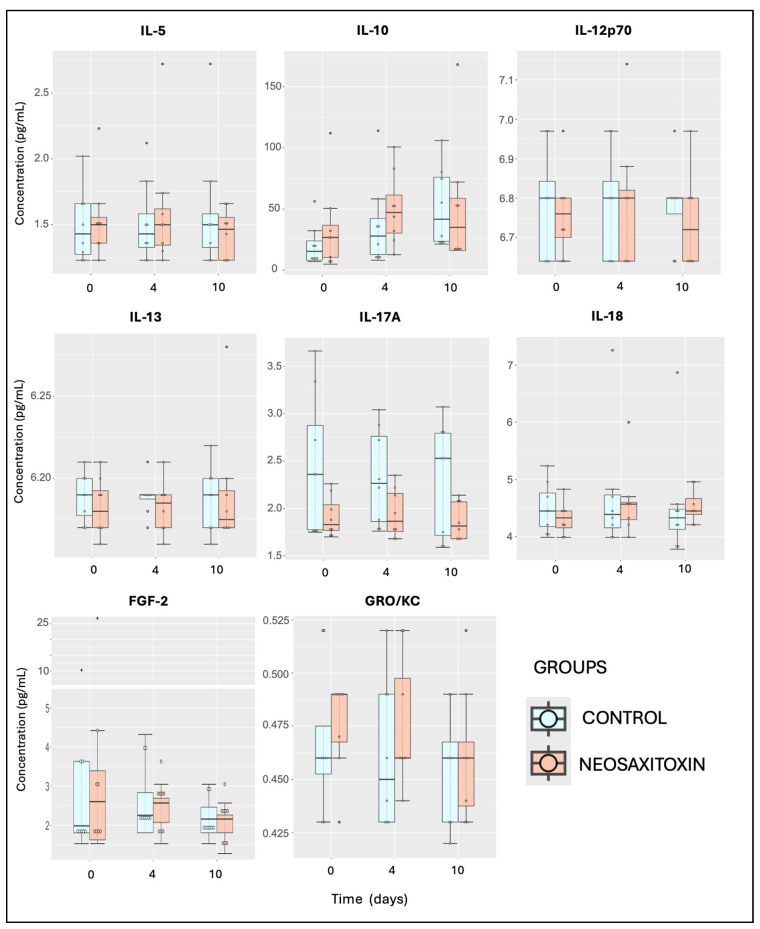
Synovial fluid cytokine and growth factor concentrations from saline- and NeoSTX-treated joints over time. Boxes represent interquartile ranges with medians and whiskers represent ranges. Individual horses are shown as colored dots. No differences were observed between groups at different time points or within groups over time. *p*-value = 0.05.

**Figure 7:**
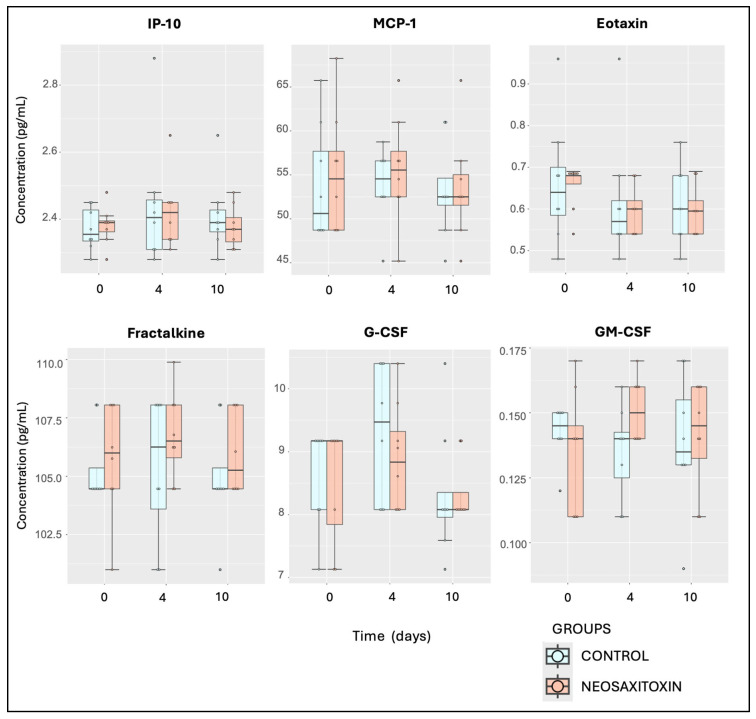
Synovial fluid chemokine concentrations from saline- and NeoSTX-treated joints over time. Boxes represent interquartile ranges with medians and whiskers represent ranges. Individual horses are shown as colored dots. No differences were observed between groups at different time points or within groups over time. *p*-value = 0.05.

Evaluations of pro-inflammatory (IFNγ, IL-1α, and IL-1β), pro-resolving (IL-10), and ambivalent cytokines and growth factors (IL-2, IL-5, IL-6, IL-8, IL-12p70, IL-13, IL-18, TNF-α, and FGF-2) showed similar values and responses in both groups, with no significant differences between joints treated with saline and those treated with NeoSTX (Figure 5 and Figure 6). Similar findings were observed for the quantification of chemokines, including eotaxin, fractalkine, G-CSF, GM-CSF, GRO/KC, IP-10, MCP-1, and RANTES, where no significant differences between joints treated with 0.9% NaCl and those treated with NeoSTX were observed (Figure 7). Nonetheless, the linear model showed a slight upregulation of IL-4 (pro-resolving cytokine) in NeoSTX-treated joints 96 h post-treatment (95% CI: 0.03 to 1.80), with this being a significant effect. The model explained 12.5% of the total variance (η^2^ = 0.125) (Table 1). Additionally, the IL-17A also showed a statistically significant effect of NeoSTX treatment when compared with the saline group, with an estimated downregulation in the outcome (95% CI: −0.73 to −0.20). No significant differences were observed between time points. The model explained 22.6% of the variance in the outcome (η^2^ = 0.226) (Table 1).

**Figure 8:**
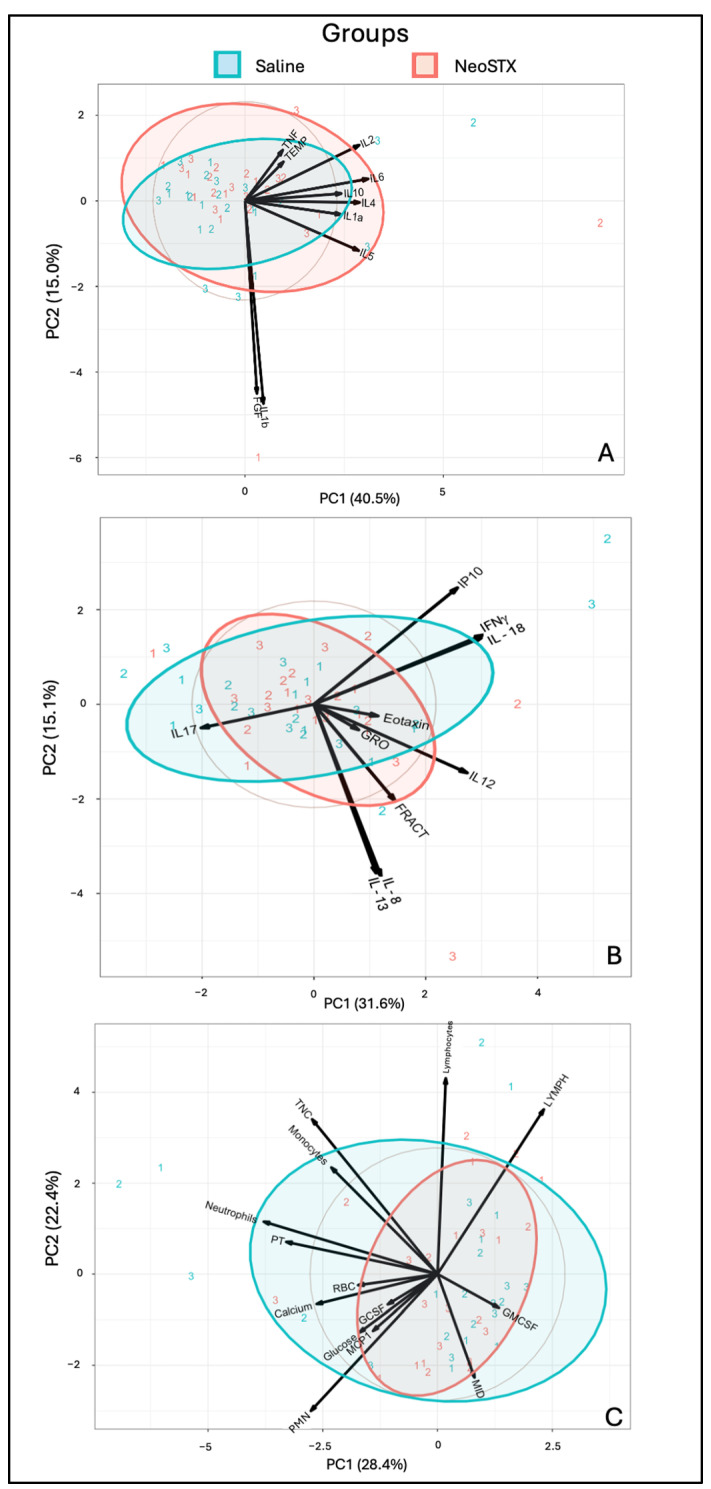
Analysis of the PC1 and PC2 representing the components capturing the major variation of the data. The clustering of both groups and at different time points shows a very similar pattern. (**A**). Representative cytokines and parameters related to joint inflammation. Samples treated with NeoSTX showed greater dispersion, particularly at time point 2, suggesting a more heterogeneous cytokine response. In contrast, saline-treated samples clustered more tightly, indicating a more uniform inflammatory profile. Cytokines such as IL-6, IL-10, IL-23, and IL-4 were strongly associated with the NeoSTX group, as evidenced by the direction and length of their loading vectors. (**B**). Principal chemokines of the synovial fluid. Key contributors to the observed variance included IP10, IFNγ, and IL-18, which were associated with the saline group, while IL-8, IL-13, IL-12, and fractalkine were more aligned with NeoSTX-treated samples. (**C**). Representation of cell population and their related chemokines in the synovial fluid. Key variables influencing group separation included total nucleated cells, neutrophils, total proteins, and calcium, which aligned more with the group. In contrast, GM-CSF, monocytes, and MCP1 were slightly more associated with NeoSTX-treated samples.

Exploratory PCA of synovial fluid biomarkers related to an inflammatory response revealed similar clustering between the NeoSTX and saline groups, primarily along PC1, which accounted for 40.5% of the variance. PC1 (40.5% of the variance) and PC2 (15.0%) together explained 55.5% of the total variability. Samples treated with NeoSTX showed greater dispersion, particularly at time point 2, suggesting a more heterogeneous cytokine response. In contrast, saline-treated samples clustered more tightly. Cytokines such as IL-6, IL-10, IL-23, and IL-4 were strongly associated with the NeoSTX group, as evidenced by the direction and length of their loading vectors (Figure 8A). Moreover, the exploratory PCA of synovial fluid chemokines also demonstrated clustering between NeoSTX and saline groups over time along PC1 (31.6% of variance) and PC2 (15.1%), together explaining 46.7% of the total variability. When interpreting these results, overlap remains. Key contributors to the observed variance included IP10, IFNγ, and IL-18, which were more associated with the saline group, while IL-8, IL-13, IL-12, and FRACT were more aligned with NeoSTX-treated samples (Figure 8B). Finally, PCA of cellular and biochemical parameters showed overlapping and similar clustering between NeoSTX and saline groups, primarily along PC1 (28.4%) and PC2 (22.4%), accounting for a combined 50.8% of the total variance. NeoSTX-treated samples clustered more tightly, suggesting a more consistent cellular response, whereas saline samples showed broader variability, especially along PC1. Key variables influencing group separation included total nucleated cells (TNCs), absolute monocyte count, absolute neutrophil count, PT, and calcium, which aligned more with the saline group. In contrast, GM-CSF, relative monocytes, and MCP1 were slightly more associated with NeoSTX-treated samples.

## 4. Discussion

This is a first-of-its-kind study that evaluated the feasibility of intra-articular administration of NeoSTX in horses. Joint injection with NeoSTX was not associated with local or systemic inflammation or short-term adverse clinical effects in healthy joints. Clinical parameters, both systemic and localized to the joint, including the assessment of lameness, surface temperature, and joint effusion, remained within normal ranges and did not differ between NeoSTX- and saline-treated groups. Similarly, synovial fluid cellular and molecular profiles, including cytokines, chemokines, and growth factor concentrations, did not differ between treatment groups or over time. Altogether, these findings suggest that intra-articular NeoSTX injection is safe and well tolerated.

Transient local inflammation, also known as joint flare, is a relatively common adverse effect observed with diagnostic anesthesia [35] and routinely used joint therapies such as hyaluronic acid [36] and orthobiologicals like mesenchymal stem cells [37] or IL-1 receptor antagonist protein [38]. Although our study included a small cohort of horses, no such adverse effects on joints were observed after the administration of a single dose of NeoSTX or 0.9% saline. No changes were evident in any of the treated joints for parameters that could have inferred active inflammatory processes (temperature, effusion, or synovial fluid cytology), as seen with local anesthetics such as lidocaine and mepivacaine following intrasynovial administration [35].

While there was a slight yet insignificant increase in surface temperature detected by thermography at 4 days, this increase was also observed in the contralateral untreated joints (of both groups) and suggested increased environmental temperature changes. Thermography was used to assess the surface temperature of the joints, being a quick and practical diagnostic tool for inflammation [39,40]. Typically, joints produce a characteristic thermal pattern, and, as a joint becomes inflamed, the thermal pattern changes [41]. Since thermography can detect subclinical inflammation even before the onset of clinical signs [40], the lack of changes in the thermal pattern of the experimental joints suggests no subclinical or clinical inflammatory effect from both NeoSTX and saline.

Synovial fluid analyses did not characterize an inflammatory response. Overall, our outcome measures were within normal ranges and reflected a short-lived, modest loss of homeostasis, typical of intra-articular administration of saline or simply arthrocentesis, that can last up to 72–96 h [42]. Sample timing was determined at 96 h and day 10 to minimize potential interference from these procedures. Furthermore, noxious inflammatory stimuli tend to result in prolonged changes in synovial fluid total protein [28,29] and concentrations of IL-1β and TNF-α (>168 h) [43]. Thus, if NeoSTX had triggered a genuine inflammatory response, we would have been able to detect it at the 96 h timepoint, as well as any delayed response at 8 or more days, considering a 72–96 h interval between samples, which was not observed in our study. These findings are like those reported when assessing the safety of autologous bone marrow mononuclear cell joint injection in normal equine tarso-crural joints [43]. However, mild early changes in synovial fluid components could have been missed. Nonetheless, other researchers reported no increase in total protein concentration following intra-articular saline administration, regardless of whether it produced an increase in cell count [44]. This was the case in our study, where the administration of 0.9% NaCl or NeoSTX did not induce an increase in synovial total protein concentration, while both treatments produced an expected short-term increase in total leukocyte count.

The administration of NeoSTX or saline produced a decrease in glucose concentration in the synovial fluid. This decrease was further intensified in the control group until day 10, while the NeoSTX group recovered its baseline values when measured on day 10. Yet, these changes failed to reach significance. A significant decrease in glucose concentration in the synovial fluid has been associated with inflammatory and/or infectious processes [30]. In our study the glucose concentration decrease observed in both groups could have been related to an early and transient inflammation response with loss of synovial fluid homeostasis induced by the arthrocentesis procedure and administration of fluid into the joint. Moreover, changes in glucose concentration observed throughout this study were within the normal reference range (Table 2) for both groups, providing more information on the non-inflammatory nature of NeoSTX.

Calcium was another metabolite evaluated to analyze the effect of NeoSTX within the joint. In this study, NeoSTX administration produced a short-term and transient decrease in calcium concentration, returning to baseline by day 10; this seemed most likely to be related to downstream NaV channel changes in calcium [15]. On the other hand, calcium concentration in the saline group remained steadily increasing until the last measurement. Nonetheless, they were within the normal range (Table 2); hence, the results obtained are most likely explained by synovial fluid transient inflammation with loss of homeostasis and not an increase in calcium concentration due to cartilage demineralization, which is often described as a result of chronic or long-term joint inflammation [31,32,48].

Several cytokines were measured in this study to ascertain NeoSTX feasibility after intra-articular administration. For this purpose, 23 biomarkers were measured, and after repeated synovial fluid sampling, no significant differences were observed between the treatment and negative control groups, supporting the innocuous effect of the toxin Ne-oSTX in a normal equine joint. It is well known that activation of the canonical nuclear factor kappa B (NF-κB) transduction pathway, probably the most important regulator pathway of pro-inflammatory gene expression, mediates the expression of pro-inflammatory cytokines such as IL-1β, IL-6, and TNF-α [20,21]. In our study, these cytokines commonly recognized as pro-inflammatory did not show significant changes in their concentration (*p* = 0.31, 0.30, and 0.44 respectively), and no significant effect size was observed at different time points (IC 95%: −0.37, 1.21; IC 95%: −0.37, 0.29; and IC 95%: −0.39, 0.96, respectively), confirming the non-inflammatory nature of NeoSTX within the joint. Other cytokines recognized as pro-resolving (IL-10), ambivalent cytokines and growth factors (IL-2, IL-5, IL-6, IL-8, IL-12p70, IL-13, IL-18, TNF-α, and FGF-2), and chemokines (G-CSF, GM-CSF, MCP-1, RANTES, Eotaxin, and IP-10) maintained their synovial fluid concentrations over the course of this study, and no significant differences were observed between groups. However, a significant effect size was observed at 4 days post-treatment in the concentration of IL-4 in the NeoSTX group (IC 95%: 0.03, 1.80), evidencing a slight upregulation of this pro-resolving cytokine. Also, there was a significant effect size in the downregulation of IL-17A (IC 95%: −0.73, −0.21). These findings suggest that NeoSTX could modulate the expression of certain cytokines differently than saline, supporting its potential immunomodulatory effects; however, further research using an inflammatory model is needed to substantiate this statement.

Although there was no significant difference, there was a statistical trend between MCP-1, G-CSF, and GM-CSF and neutrophils and monocytes at day 4 for both groups. This cellular response followed an expected pattern according to the specific function of these chemokines, in response to arthrocentesis and joint injection [49,50,51]. Taking together the information gathered in this study, it is possible to discern that the results refer only to the administration of saline solution and the arthrocentesis procedure rather than an inflammatory process per se. These findings support the innocuous effect of NeoSTX and suggest that the intra-articular use of NeoSTX is safe.

Sample size is always a limitation of studies using large animals in comparison to murine models. While our sample size is the typical sample size for equine and translational large animal orthopaedic models, we nonetheless acknowledge that some actual differences could have been missed due to the study potentially being underpowered as a result of the small sample size and sex imbalance between groups. Another limitation of this study is that we were unable to access a controlled temperature room for thermographic data gathering, which could have affected the temperatures obtained during this study; nonetheless, we were aware of this situation, and we took all the measurements at the same time over the days to decrease the measurement bias. The above limitation was somewhat minimized by measuring other clinical parameters such as joint effusion and locomotor soundness. Assessing cartilage metabolism biomarkers, cartilage histology, and synovial membrane could have further elucidated the safety of the drug.

## 5. Conclusions

Based on the results obtained, along with previously available information, we can conclude that intra-articular administration of 20 µg of NeoSTX is feasible and without systemic or joint adverse effects in the short term. Ongoing studies will elucidate the ability of Neo-STX to modulate inflammatory response in the treatment of osteoarthritis.

## Figures and Tables

**Table 1:** Summary of effect sizes (η^2^) and 95% confidence intervals of ANOVA results obtained from the analysis of synovial fluid biomolecules and cell populations at different time points. The linear model revealed a statistically significant effect at day 4 post-treatment for the absolute monocyte counts (IL-4), explaining approximately 12% of the total variance in the outcome for both parameters. Additionally, the model also revealed a significant effect size for IL-17A, explaining approximately 23% of the total variance in the outcome. As the confidence interval did not include zero, this indicated a statistically significant effect.

Parameter	Size Effect (η^2^)	95% Confidence Interval
Calcium	0.016	[−1.38, 1.57]
Glucose	0.029	[−7.40, 15.03]
Total Protein	0.039	[−0.91, 0.27]
Red Blood Cells	0.046	[−0.026, 0.087]
WBC (×10^9^/L)	0.076	[−1.30, 0.48]
Lymphocytes (×10^9^/L)	0.078	[−0.39, 0.53]
Monocytes (×10^9^/L)	0.066	[−0.23, 0.11]
Neutrophils (×10^9^/L)	0.045	[−0.88, 0.24]
Lymphocytes (%)	0.077	[−9.89, 15.87]
Monocytes (%)	0.12	[1.11, 11.96]
Neutrophils (%)	0.029	[−15.44, 9.44]
IL-1a	0.066	[−0.64, 2.36]
IL-1b	0.045	[−0.37, 1.21]
IL-2	0.052	[−0.47, 0.55]
IL-4	0.125	[0.03 to 1.80]
IL-5	0.004	[−0.21, 0.19]
IL-6	0.053	[−0.37, 0.29]
IL-8	0.051	[−1.16, 2.76]
IL-10	0.109	[−11.06, 28.93]
IL-12p70	0.012	[−0.09, 0.05]
IL-13	0.013	[−0.015, 0.009]
IL-17A	0.23	[−0.73, −0.21]
IL-18	0.03	[−0.49, 0.29]
TNF-a	0.052	[−0.39, 0.96]
FGF-2	0.079	[−1.49, 2.84]
GRO/KC	0.069	[−0.007, 0.027]
IFN-g	0.055	[−3.39, 1.71]
IP10	0.052	[−0.08, 0.05]
MCP-1	0.020	[−2.31, 4.49]
Eotaxin	0.067	[−0.07, 0.04]
Fractalkine	0.063	[−0.33, 2.24]
G-CSF	0.134	[−0.67, 0.35]
GM-CSF	0.019	[−0.007, 0.015]
RANTES	0.031	[−0.002, 0.002]

**Table 2:** Mean values of total proteins, calcium, and glucose in both treatment groups at different time points and the reference values for equines.

Parameter	Group	Pre-Treatment (Mean ± sd)	4 d. (Mean ± sd)	10 d. (Mean ± sd)	Reference Values
Total Protein (mg/dL)	NeoSTX	1.88 ± 0.82	1.61 ± 0.72	1.83 ± 0.71	<2.5 g/dL [45]
	Control	2.16 ± 1.33	1.94 ± 1.22	2.19 ± 1.22	
Ca^2+^ (mg/dL)	NeoSTX	10.1 ± 2.29	8.71 ± 1.67	9.30 ± 0.9	8.3–10.7 [46]
	Control	8.83 ± 1.98	9.10 ± 2.19	9.93 ± 4.63	
Glucose (mg/dL)	NeoSTX	104.16 ± 13.4	96.9 ± 20.4	104.28 ± 23.0	33–105 mg/dL [47]
	Control	102.55 ± 17.7	96.9 ± 22.7	95.26 ± 18.9	

## Data Availability

The original contributions presented in this study are included in the article/Supplementary Material. Further inquiries can be directed to the corresponding author.

## Supplementary Materials

The following supporting information can be downloaded at https://www.mdpi.com/article/10.3390/ani15162453/s1, Table S1. GLMM analysis and post-hoc pairwise comparison results of biomarker of each group and their 95% Confidence interval. Baseline pre-treatment. Table S2. GLMM analysis and post-hoc pairwise comparison results of biomarker of each group and their 95% Confidence interval. 4 days post-treatment. Table S3. GLMM analysis and post-hoc pairwise comparison results of biomarker of each group and their 95% Confidence interval. 10 days post-treatment.
