# Supplementary material for: Synovial Fluid Biomarker Profile After Intra-Articular Administration of Neosaxitoxin in Horses: A Feasibility Study
Source: Animals (Basel). 2025 Aug 21;15(16):2453. doi: 10.3390/ani15162453 (PMC12382866; doi:10.3390/ani15162453)
Supplement: Supplementary file 1 [file animals-15-02453-s001.zip › animals-3763324-supplementary.pdf]

Parameter	Treatment	Pairwise contrast (p-value)	95% Confidence Interval
Calcium	NeoSTX	0.31	[8.31, 12.00]
	Saline		[7.01, 10.70]
Glucose	NeoSTX	0.79	[90.2, 119]
	Saline		[87.7, 116]
Total Protein	NeoSTX	0.59	[1.13, 2.63]
	Saline		[1.41, 2.91]
Red Blood Cells	NeoSTX	0.64	[-0.019, 0.12]
	Saline		[-0.042, 0.097]
WBC (x10^9/L)	NeoSTX	0.54	[0.001, 2.32]
	Saline		[0.59, 2.91]
Lymphocytes (x10^9/L)	NeoSTX	0.78	[0.11, 1.29]
	Saline		[0.22, 1.40]
Monocytes (x10^9/L)	NeoSTX	0.75	[0.016, 0.44]
	Saline		[-0.026, 0.40]
Neutrophils (x10^9/L)	NeoSTX	0.29	[-0.49, 0.95]
	Saline		[0.03, 1.47]
IL-1α	NeoSTX	0.78	[1.69, 5.42]
	Saline		[1.34, 5.07]
IL-1β	NeoSTX	0.82	[3.45, 4.06]
	Saline		[3.50, 4.11]
IL-2	NeoSTX	0.95	[1.55, 2.86]
	Saline		[1.53, 2.84]
IL-4	NeoSTX	0.64	[40.9, 42.7]
	Saline		[40.6, 42.4]
IL-5	NeoSTX	0.77	[1.29, 1.80]
	Saline		[1.24, 1.75]
IL-6	NeoSTX	0.86	[0.06, 0.89]
	Saline		[011, 0.94]
IL-8	NeoSTX	0.69	[8.44, 13.1]
	Saline		[7.79, 12.5]
IL-10	NeoSTX	0.44	[8.60, 59.6]
	Saline		[-5.07, 46.0]
IL-12p70	NeoSTX	0.74	[6.67, 6.86]
	Saline		[6.69, 6.88]
IL-13	NeoSTX	0.56	[6.16, 6.19]
	Saline		[6.17, 6.20]
IL-17A	NeoSTX	**0.027***	[1.57, 2.26]
	Saline		[2.12, 2.81]
IL-18	NeoSTX	0.58	[3.83, 4.28]
	Saline		[4.02, 5.01]
TNF-α	NeoSTX	0.89	[-0.273, 1.38]
	Saline		[-0.195, 1.46]
FGF-2	NeoSTX	0.25	[2.83, 8.20]
	Saline		[0.64, 6.01]
GRO/KC	NeoSTX	0.56	[0.46, 0.49]
	Saline		[0.45, 0.49]
IFN-γ	NeoSTX	0.43	[51.5, 57.7]
	Saline		[53.0, 59.5]
IP10	NeoSTX	0.85	[2.30, 2.46]
	Saline		[2.29, 2.45]
MCP-1	NeoSTX	0.66	[50.8, 59.5]
	Saline		[49.5, 58.2]
Eotaxin	NeoSTX	0.86	[0.58, 0.73]
	Saline		[0.59, 0.74]
Fractalkine	NeoSTX	0.72	[104, 107]
	Saline		[104, 107]
G-CSF	NeoSTX	0.79	[7.89, 9.16]
	Saline		[8.01, 9.28]
GM-CSF	NeoSTX	0.42	[0.121, 0.149]
	Saline		[0.129, 0.156]

Table S1. GLMM analysis and post-hoc pairwise comparison results of biomarker of each group and their 95% Confidence interval. Baseline pre-treatment.

Table S2. GLMM analysis and post-hoc pairwise comparison results of biomarker of each group and their 95% Confidence interval. 4 days post-treatment.

Parameter	Treatment	Pairwise contrast (p-value)	95% Confidence Interval
Calcium	NeoSTX	0.76	[6.89, 10.54]
	Saline		[7.28, 10.92]
Glucose	NeoSTX	0.99	[82.5, 111]
	Saline		[82.5, 111]
Total Protein	NeoSTX	0.53	[0.86, 2.36]
	Saline		[1.18, 2.69]
Red Blood Cells	NeoSTX	0.74	[-0.037, 0.10]
	Saline		[-0.021, 0.12]
WBC (x10^9/L)	NeoSTX	0.49	[0.58, 2.90]
	Saline		[1.12, 3.44]
Lymphocytes (x10^9/L)	NeoSTX	0.60	[0.36, 1.53]
	Saline		[0.15, 1.33]
Monocytes (x10^9/L)	NeoSTX	**0.044***	[0.011, 0.43]
	Saline		[0.31, 0.74]
Neutrophils (x10^9/L)	NeoSTX	0.37	[-0.16, 1.29]
	Saline		[0.29, 1.74]
IL-1α	NeoSTX	0.11	[3.49, 7.21]
	Saline		[1.33, 5.06]
IL-1β	NeoSTX	0.86	[3.34, 3.96]
	Saline		[3.38, 3.99]
IL-2	NeoSTX	0.86	[2.05, 3.36]
	Saline		[1.98, 3.28]
IL-4	NeoSTX	0.086	[42.2, 44.0]
	Saline		[41.1, 42.9]
IL-5	NeoSTX	0.57	[1.36, 1.87]
	Saline		[1.26, 1.77]
IL-6	NeoSTX	0.76	[0.44, 1.27]
	Saline		[0.35, 1.18]
IL-8	NeoSTX	0.38	[7.46, 12.2]
	Saline		[8.93, 13.6]
IL-10	NeoSTX	0.46	[24.68, 75.7]
	Saline		[11.78, 62.8]
IL-12p70	NeoSTX	0.88	[6.70, 6.89]
	Saline		[6.69, 6.88]
IL-13	NeoSTX	0.56	[6.176, 6.207]
	Saline		[6.173, 6.204]
IL-17A	NeoSTX	0.12	[1.60, 2.29]
	Saline		[1.98, 2.67]
IL-18	NeoSTX	0.76	[4.12, 5.11]
	Saline		[4.22, 5.22]
TNF-α	NeoSTX	0.95	[-0.220, 1.43]
	Saline		[-0.187, 1.47]
FGF-2	NeoSTX	0.97	[-0.199, 5.17]
	Saline		[-0.13, 5.24]
GRO/KC	NeoSTX	0.33	[0.45, 0.49]
	Saline		[0.44, 0.48]
IFN-γ	NeoSTX	0.84	[54.1, 60.6]
	Saline		[53.7, 60.2]
IP10	NeoSTX	0.75	[2.34, 2.50]
	Saline		[2.36, 2.52]
MCP-1	NeoSTX	0.58	[51.2, 59.9]
	Saline		[49.6, 58.2]
Eotaxin	NeoSTX	0.70	[0.52, 0.67]
	Saline		[0.54, 0.69]
Fractalkine	NeoSTX	0.22	[105, 108]
	Saline		[104, 107]
G-CSF	NeoSTX	0.38	[8.27, 9.54]
	Saline		[8.66, 9.93]
GM-CSF	NeoSTX	0.10	[0.137, 0.165]
	Saline		[0.121, 0.149]

Table S3. GLMM analysis and post-hoc pairwise comparison results of biomarker of each group and their 95% Confidence interval. 10 days post-treatment.

**Parameter**	**Treatment**	**Pairwise contrast (p-value)**	**95% Confidence Interval**
Calcium	NeoSTX	0.62	[7.47, 11.13]
	Saline		[8.10, 11.75]
Glucose	NeoSTX	0.37	[89.6, 118]
	Saline		[80.7, 109]
Total Protein	NeoSTX	0.49	[1.08, 2.59]
	Saline		[1.44, 2.94]
Red Blood Cells	NeoSTX	0.083	[0.042, 0.18]
	Saline		[-0.044, 0.10]
WBC (x10^9/L)	NeoSTX	0.91	[-0.1, 2.22]
	Saline		[-0.005, 2.32]
Lymphocytes (x10^9/L)	NeoSTX	0.79	[-0.19, 0.98]
	Saline		[-0.29, 0.88]
Monocytes (x10^9/L)	NeoSTX	0.54	[0.14, 0.56]
	Saline		[0.05, 0.48]
Neutrophils (x10^9/L)	NeoSTX	0.97	[-0.11, 1.34]
	Saline		[-0.12, 1.33]
IL-1α	NeoSTX	0.95	[1.30, 5.02]
	Saline		[1.21, 4.94]
IL-1β	NeoSTX	0.18	[3.36, 3.97]
	Saline		[3.65, 4.26]
IL-2	NeoSTX	0.94	[1.88, 3.18]
	Saline		[1.84, 3.15]
IL-4	NeoSTX	0.88	[41.2, 43.0]
	Saline		[41.1, 42.9]
IL-5	NeoSTX	0.33	[1.18, 1.69]
	Saline		[1.35, 1.87]
IL-6	NeoSTX	0.57	[0.14, 0.97]
	Saline		[0.30, 1.13]
IL-8	NeoSTX	0.057	[11.11, 15.8]
	Saline		[7.89, 12.6]
IL-10	NeoSTX	0.99	[26.07, 77.1]
	Saline		[25.85, 76.9]
IL-12p70	NeoSTX	0.54	[6.65, 6.84]
	Saline		[6.69, 6.88]
IL-13	NeoSTX	0.72	[6.176, 6.207]
	Saline		[6.172, 6.203]
IL-17A	NeoSTX	0.059	[1.53, 2.21]
	Saline		[1.99, 2.68]
IL-18	NeoSTX	0.95	[4.04, 5.03]
	Saline		[4.06, 5.05]
TNF-α	NeoSTX	0.11	[0.724, 2.38]
	Saline		[-0.238, 1.42]
FGF-2	NeoSTX	0.96	[-0.56, 4.81]
	Saline		[-0.47, 4.89]
GRO/KC	NeoSTX	0.68	[0.44, 0.48]
	Saline		[0.43, 0.47]
IFN-γ	NeoSTX	0.59	[53.7, 60.2]
	Saline		[54.9, 61.4]
IP10	NeoSTX	0.54	[2.30, 2.46]
	Saline		[2.33, 2.49]
MCP-1	NeoSTX	0.92	[49.2, 57.9]
	Saline		[48.9, 57.6]
Eotaxin	NeoSTX	0.81	[0.52, 0.67]
	Saline		[0.54, 0.69]
Fractalkine	NeoSTX	0.34	[104, 108]
	Saline		[103, 107]
G-CSF	NeoSTX	0.95	[7.72, 8.99]
	Saline		[7.69-8.96]
GM-CSF	NeoSTX	0.79	[0.127, 0.155]
	Saline		[0.125, 0.153]

Randomization and Blinding Procedures:

a) A simple randomization method was employed to assign treatments to horses in sequential order (1 to 16), ensuring an equal number of animals per treatment group. Subsequently, either the left or right joint was randomly selected for treatment, with the contralateral joint serving as the untreated control. Randomization was completed prior to the selection and arrival of the horses, ensuring allocation concealment.

b) Following treatment administration, only the principal investigator (CD) was aware of the assigned treatments. Clinical parameters were assessed and recorded by a separate, blinded evaluator who had been trained before the start of the study.

c) All biomarker assays were conducted in a blinded manner by an independent external laboratory. The laboratory personnel received only alphanumeric codes with no identifying information related to the study.

d) Importantly, due to the anatomical characteristics of the equine joints, all intended samples were successfully obtained and analyzed. The statistical analysis plan was pre-registered prior to data collection.
